# AAV-mediated gene therapy: Advancing cardiovascular disease treatment

**DOI:** 10.3389/fcvm.2022.952755

**Published:** 2022-08-19

**Authors:** Huili Zhang, Qi Zhan, Biao Huang, Yigang Wang, Xiaoyan Wang

**Affiliations:** ^1^College of Life Sciences and Medicine, Zhejiang Sci-Tech University, Hangzhou, China; ^2^Oncology Department, Zhejiang Xiaoshan HospitaI, Hangzhou, China

**Keywords:** AAV, gene therapy, CVD, cardiomyocyte, heart failure

## Abstract

Gene therapy has revolutionized the field of medicine, offering new hope for those with common and rare diseases. For nearly three decades, adeno-associated virus (AAV) has shown significant therapeutic benefits in multiple clinical trials, mainly due to its unique replication defects and non-pathogenicity in humans. In the field of cardiovascular disease (CVD), compared with non-viral vectors, lentiviruses, poxviruses, and adenovirus vectors, AAV possesses several advantages, including high security, low immunogenicity, sustainable and stable exogenous gene expression etc., which makes AAV one of the most promising candidates for the treatment of many genetic disorders and hereditary diseases. In this review, we evaluate the current information on the immune responses, transport pathways, and mechanisms of action associated with AAV-based CVD gene therapies and further explore potential optimization strategies to improve the efficiency of AAV transduction for the improved safety and efficiency of CVD treatment. In conclusion, AAV-mediated gene therapy has great potential for development in the cardiovascular system.

## Introduction

According to data provided by The Global Burden of Diseases, Injuries, and Risk Factors Study in 2019, cardiovascular disease (CVD) is one of the leading causes of deaths, accounting for 18.5 million deaths, i.e., about one third of all mortalities worldwide, making it a major public health issue, with China having the largest number of cardiovascular disease deaths (1). This suggests that, in the future, providing both CVD prevention and treatment interventions in the context of sustainable infrastructure development may play important roles in global CVD control.

Since the successful engraftment of β-galactosidase-expressing endothelial cells into denuded iliofemoral arteries in 1989, gene therapy has quickly emerged as a candidate for the treatment of CVD (2). In the 1970s, Friedmann and Roblin formally proposed the concept of gene therapy (3): a strategy to treat diseases by means of non-viral or viral vectors that introduces therapeutic exogenous genes into target cells and tissues to supplement or correct defective genes through *in vitro* and *in vivo* techniques (4, 5). However, due to barriers to the effective delivery of gene fragments or gene-editing tools in the body, the development of gene therapy was slow until scientists discovered that some viruses seemed to be able to overcome these barriers. Virus surface proteins effectively recognize cell receptors and carry genetic material into the host cell, where it is continuously produced by the host's cell factories, making viruses ideal delivery vehicles for transgenes (6–8). Therefore, the application of viral vectors has ushered in key advancements in the conceptual exploration and clinical application of gene therapy.

Adeno-associated virus (AAV) vectors have attracted widespread attention because of their unique advantages in effectively, persistently, and safely expressing foreign genes in host cells with low immunogenicity (9). Over the past 30 years, recombinant (r) AAV products have been successfully adopted for the clinical treatment of a variety of diseases, including cancers (10), ophthalmic diseases (11), blood diseases, neurological diseases, and immune system diseases among others (12). To date, three drugs based on rAAV vectors have been approved for marketing. The first rAAV-based drug, Glybera (Alipogene Tiparvovec, AT), which was approved by the European Union, is an AAV1 vector drug that delivers DNA encoding functional lipoprotein lipase (LPL) to skeletal muscle for patients with LPL deficiency due to gene mutations (13), but it was taken off the market soon after its release because the price was unexpectedly high. In 2017, Luxturna was the first AAV-based ophthalmic gene therapy drug approved by the FDA and is used for the symptomatic treatment of retinal dystrophy associated with double-copy RPE65 mutations (14). In 2019, Zolgensma was the second AAV-based drug approved by the FDA and utilizes AAV9 expressing a functional SMN1 transgene to treat spinal muscular atrophy type I (15).

Together, conditions of the cardiovascular system can broadly be classified into two main categories: heart diseases and vascular diseases. It is encouraging that the development of modern molecular biology has prompted more thorough research on CVD, focusing on the overexpression, knockdown, and knockout of key pathogenic genes. Such genetic manipulation requires advances in technology or transmitters, hence the emergence of viral vectors that can act as delivery vehicles may lead to new insights. From the vast sea of viruses, preceding research has screened out the three most commonly used virus tools for CVD, namely, HIV-1, adenovirus, and parvovirus (e.g., AAV), which have the following common characteristics: (1) high infection efficiency; (2) low cytotoxicity; and (3) strong expression effects (8). Among them, rAAV vectors have become the best choice for gene therapy of CVD due to their unique advantages.

In recent years, with the development of high-throughput genetic engineering, cell therapy, and immunotherapy, research on CVD has become increasingly in-depth. Currently, many clinical studies have shown the impressive benefits of AAV-based therapies, 18 studies of rAAVs-based CVD gene therapies have been documented (from https://clinicaltrials.gov/; keywords, adeno associated virus/interventional studies/CVD). We provide a detailed overview of these preclinical and clinical studies, systematically introduce the optimized strategies being used to develop AAV as gene delivery vectors with improved specific targeting capabilities, and discuss the prospects of and potential research into using this vector system in the rapidly evolving field of CVD treatment.

## Systematic characteristics of AAV and rAAV vectors

### Composition, configuration, and affinity of AAV vectors

**Figure 1:**
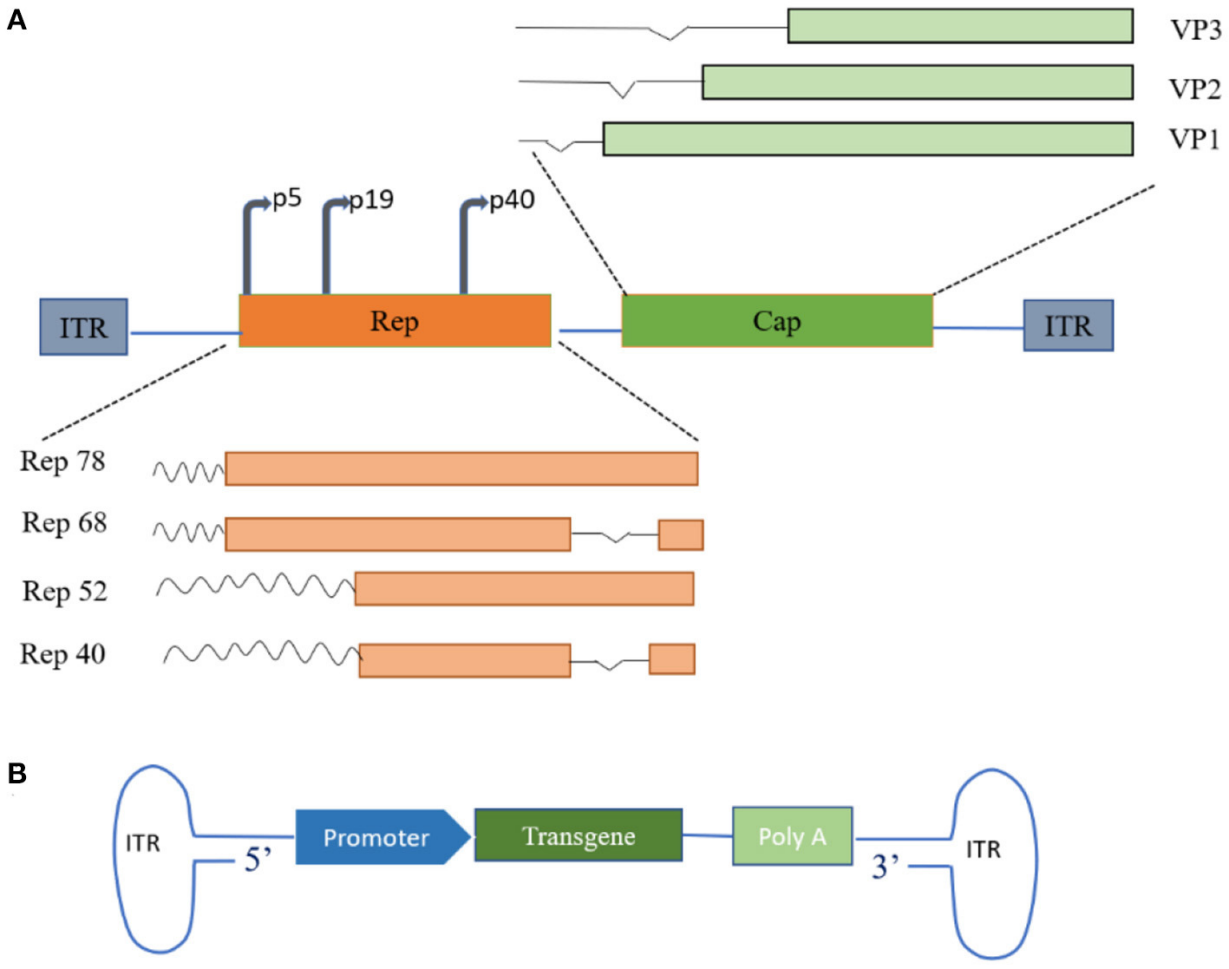
Schematic of adeno-associated virus (AAVs) genome structure. **(A)** Wild-type AAV genome structure. The wild-type AAV comprises a small, unenveloped, single-stranded DNA genome of approximately 4.7 Kb, encoding replication (Rep) and capsid (Cap) genes. Rep and Cap protein products are assembled into 60-polymeric viral capsids under suitable conditions. **(B)** Recombinant AAV genome structure. Therapeutic transgenes replace parts of the genome encoding proteins of the viral capsid. ITR, inverted terminal repeat; VP, capsid proteins.

AAV is an icosahedral parvovirus that cannot replicate without the aid of host cell machinery or helper viruses. It was first described as a contaminating component of purified adenovirus preparations in the mid-1960s and was soon discovered in human tissues (16). AAV is one of the most actively developed vectors for cell and gene therapy applications and has contributed to encouraging results in clinical research into a variety of human diseases. AAV is a 4.7 Kbp single-stranded DNA virus whose genome consists of Rep and Cap genes flanked by inverted terminal repeats (ITRs), each consisting of 145 bases (4, 10, 17). Two promoters, p5 and P19, on the left side of the AAV genome transcribe mRNAs of different lengths and generate four different capsid proteins containing overlapping sequences (Rep78, Rep68, Rep52, and Rep40) by selective shearing (4, 18). The capsid genes encode three capsid proteins (VP1, VP2, VP3) and assembly activator protein (AAP) through the alternative splicing of different start codons (19), and the proportion of the three structural proteins in a mature virion is approximately 1:1:10. The viral capsid serves as the vehicle for viral gene delivery (Figure 1) (12). In general, replication and efficient infection only occur with the involvement of helper adenoviruses or herpes simplex virus. Interestingly, AAV can establish latency in the absence of helper viruses. In the latent state, Rep mediates the activation of AAVS1-specific DNA synthesis at human chromosome 19q13.4qter and the formation of integrated complexes *in vitro* and *in vivo* (20, 21).

The rAAV vectors currently in use worldwide are constructed by replacing a section of the viral genome between the two ITRs with a genetic payload of interest, and rAAVs have shown some unique advantages in clinical trials compared to wild-type AAVs (14, 19, 22). The development of rAAV vectors has received overwhelming enthusiasm from the research community. It is generally believed that the key to gene therapy is the unification of specificity and controllability. AAV has a simple structure, and 13 human and primate AAV serotypes and hundreds of AAV mutants have been described (23–25). In addition, different serotypes of AAV can infect diverse cell types, depending on the receptors on the cell surface targeted by AAV, and these serotypes and their associated receptors also determine the transport routes taken by the different AAV serotypes within the cell (Table 1). With the development of high-throughput biotechnology, an increasing variety of specific AAV serotypes are being developed, and this has largely driven the application of AAV as agents to fight human disease.

**Table 1:** Receptor and tissue selectivity of AAV serotypes (4, 26–31).

**AAV serotype**	**Primary receptor**	**Co-receptor**	**Tissue selectivity**
AAV1	α2-3/α2-6 N linked SA	AAVR, α5β1 integrin	CNS, heart, liver, lung, kidney, eye
AAV2	HS	AAVR, FGFR1, HGFR, LamR, integrin, c-MET	CNS, liver, muscle, kidney, eye, brain, eye
AAV3	HS	AAVR, FGFR, HGFR, LamR, c-MET	Muscle, liver
AAV4	α2-3 O-Linked SA	Unknown	CNS, eye, heart
AAV5	α2-3 N linked SA	AAVR, PDGFR	CNS, eye, lung, liver, kidney
AAV6	HS, α2-3/α2-6 N linked SA	AAVR, EGFR	CNS, heart, lung, muscle
AAV7	Unknown	α5β1 integrin	CNS, muscle, liver
AAV8	LamR	AAVR, α5β1 integrin	CNS, heart, liver, skeletal muscle, pancreas, eye, retinal, brain
AAV9	N-linked galactose	AAVR, LamR, α5β1 integrin	CNS, heart, muscle, liver, kidney, lung, pancreas, retinal, brain, testes
AAV10	Unknown	α5β1 integrin	Muscle, liver
AAV11	Unknown	Unknown	Unknown
AAV12	Unknown	Unknown	Nasal
AAV13	HS	Unknown	Liver

AAV, adeno-associated virus; SA, sialic acid; AAVR, adeno-associated virus receptor; CNS, central nervous system; HS, heparan sulfate; FGFR1, fibroblast growth factor receptor 1; HGFR, hepatocyte growth factor receptor; LamR, laminin receptor 1; c-MET, human hepatocyte growth factor; PDGFR, platelet-derived growth factor receptor; EGFR, epidermal growth factor receptor

### AAV-vector-mediated transduction and related challenges

#### AAV-vector-mediated transduction

**Figure 2:**
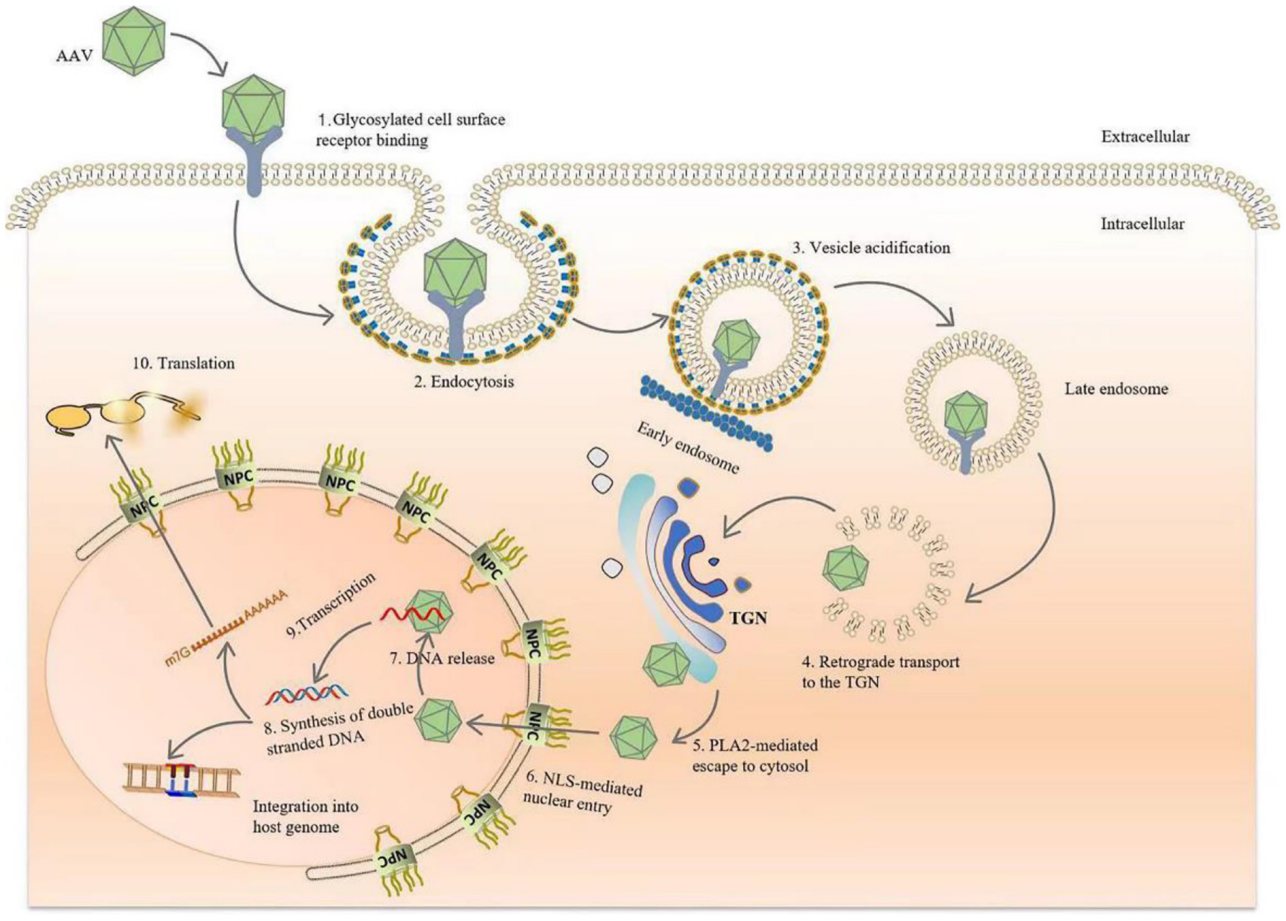
Diagram of AAV transduction mechanism. AAV is bound by glycosylated receptors on the surface of host cells, initiating endocytosis, after which it is rapidly transported to the reverse Golgi apparatus (TGN). After arriving at the TGN, the conformations of VP1 and VP2 change due to the acidification of endocytic vesicles in the lower intracellular pH environment, and the phospholipase A2 domain of the capsid species is exposed. After endosomal escape, AAV crosses the Golgi membrane and enters the nucleus via a nuclear pour complex (NPC) guided by the nuclear localization sequence (NLS). Once in the nucleus, DNA is released from the capsid, and host cell DNA polymerase is used to rapidly initiate second-strand synthesis. Finally, viral genomes persistently express the transgenes. Vector genomes also integrate into the host genome to establish latency, although the probability of this event occurring is extremely low.

The viral capsid protein significantly affects the affinity of the AAV vector for a given tissue. *In vitro* studies support evidence that the transduction of AAV to cells requires AAV capsid protein interactions with primary receptors and co-receptors on the target cell surface. AAV enters the target cell, initiates a cascade of reactions, and finally, integrates DNA into the target cell chromosome (without helper virus) or otherwise persistently expresses viral genes *via* additional methods (with helper virus present) (4, 16, 23, 26, 27). In conclusion, during natural infection, AAV relies on its capsid proteins attaching to glycans expressed on the surface of target cells, such as heparan sulfate (AAV2, 3, 6, 13), sialic acid (AAV1,4,5,6), laminin receptor (AAV8), and galactose (AAV9) (16, 22). Subsequently, the virus undergoes internalization and intracellular transport through endocytosis, then escapes from the endosome under the control of the capsid protein while completing nuclear import, releasing the viral genome. Once the DNA is released from the capsid, the single-stranded genome is immediately synthesized and converted into double-stranded DNA (Figure 2) (4, 16, 22, 26).

When AAV successfully infects a cell, it determines the final location of its genes based on the presence or absence of helper viruses. In the absence of helper viruses, AAV is nearly incapable of replication, gene expression is suppressed, and the AAV genome integrates into a 4 kb region of chromosome 19q13.4, establishing a latent infection (20, 21). With a helper virus, AAVs can undergo nucleic acid replication, viral gene expression, virion production, and ultimately, virulent infection (14, 32). Interestingly, rAAV rarely integrates DNA into the genome of the target cell, and thus largely avoids the likelihood of inadvertent genome insertion (15).

The transduction efficiency of AAV is critical for AAV-based gene therapy, and several approaches used to improve this efficiency have emerged. Treatment of AAV with the metal ions cobalt and zinc increased the transduction efficiency of the AAV vector 10-fold in a dose-dependent manner (33). Most worthy of mention are the modification and engineering of the AAV capsid and the remodeling strategy of the promoter, which will be highlighted later.

The gene delivery approaches of AAV in CVD can be mainly summarized as follows. The most common is systemic intravenous delivery, which is simple but relatively unspecific. The second is intramyocardial delivery, which has the advantages of strong specificity and no endothelial barrier but causes greater damage (34). Anterograde/retrograde coronary injection and pericardial injection have also been selected as routes of AAV delivery for CVD (34). Intravenous and coronary injections are commonly used as delivery methods in clinical studies because maximizing patient safety is the primary clinical concern (34, 35).

#### Innate challenges of AAV vectors

Currently, AAV is one of the most promising vector platforms for *in vivo* gene transfer due to its unique advantages. However, immune recognition of AAVs is a potential challenge to its therapeutic efficacy. Early preclinical and clinical trials have demonstrated the high prevalence of neutralizing antibodies (NAbs) in the global population due to the natural exposure of humans and non-human primates to wild-type AAV (23, 36–38). These pre-existing human host immune responses potentially inhibit the efficiency of rAAV gene therapy. To date, various strategies have been developed to address the challenges raised by NAbs, all with encouraging success. Chemical modification of capsids alter the surface of AAV to facilitate their evasion of immunological detection (39). The administration of empty capsids, which act as bait to neutralize NAbs, leads to AAV vectors carrying exogenous payloads infecting target tissues efficiently and accurately (40, 41). IgG-cleaving enzyme modification by IdeS or IdeZ also offers a potential way to rescue gene transfer of the AAV vector by removing pre-existing anti-AAV antibodies and prevent AAV neutralization *in vivo* (42, 43). In non-human primate experiments, the transient removal of pre-existing AAV vector NAbs *in vivo* using plasmapheresis allowed the sustained expression of high levels of AAV-vector-delivered transgenes (44, 45). Notably, Orlowski et al. used apheresis and AAV9 particles coupled to agarose beads to selectively deplete pre-existing carrier-bound antibodies *in vivo*. Strategically, this depletion of anti-AAV antibodies was specific, and the immunoadsorption technique greatly increased the AAV transduction rate in the heart and liver of animals (46). Taken together, these strategies circumvent the deleterious effects of anti-AAV antibodies to a certain extent and can significantly improve and enhance the gene therapy efficacy of rAAVs.

#### Safety of rAAVs vectors

The rAAV-mediated gene management of patients with refractory or relapsing CVD has been the subject of extensive research. Although efficacy has been demonstrated in preclinical studies and in phase 1 and 2 trials, there is no clear evidence of efficacy in large randomized placebo-controlled trials. What is worrying, however, is that, in a recent clinical study, 9 AAV-cFVIII-treated hemophilia A dogs continued to express coagulation factors for 10 years of follow-up. Moreover, the therapeutic gene segment carried by AAV was partially integrated into the host cell chromosome, especially in the vicinity of genes related to cell growth, which has the potential to induce cancer, although the gene integration rate was extremely low (47). In addition, a European study found that AAV DNA was detectable in 21% of patients, and the main viral subtypes were AAV2 and AAV2/13 (28). This study shatters the myth that AAV viral vectors are absolutely safe. A 46-nt liver-specific enhancer-promoter element in the wild-type AAV2 genome has been reported to be associated with the dysregulation of human hepatocellular carcinoma (HCC) driver genes (48). Schäffer et al. integrated AAV2/13 into the genomes of Thai and Mongolian HCC patients and found that they did not induce tumorigenesis (49). Overall, the very few potential gene-integration risks of AAV do not invalidate the advantages of AAV (50, 51). This is because AAV remains, mainly in its episomal form, in non-dividing mature cells and is still considered a safe vector choice compared to retroviruses that integrate into genomes.

### Gene modification to achieve AAV-specific targeting

#### Take-off cardiovascular system—rational selection of AAV serotypes

Different serotypes of AAV have diverse affinity for tissues or target organs. The difference between the 13 native serotypes and hundreds of wild-type variants lies in the sequence variation of the Cap gene, which encodes individual capsid proteins, resulting in the variable infection efficiency, tissue affinity, and expression times of the AAV serotypes in different cells and tissues (14, 26, 40). Among these serotypes, AAV1, 6, 8, and 9 are considered the most favorable candidates for specific cardiac transduction (52–55). AAV9 has the best cardiac transduction specificity and can be expressed stably and with high efficiency. AAV1, 5, 8, and 9 have good transduction specificity for blood vessels. In recent years, new methods to screen and obtain more targeted rAAV vectors have been implemented, such as point mutation of capsid proteins to obtain AAV variants and the introduction of new peptides into capsid proteins. A reversible RNA switch hammerhead ribozyme was reported to efficiently regulate transgene expression *in vivo* without involving immunogenic non-self proteins, broadening the application dimensions of AAV gene therapy (56). Anc80L65, a synthetic AAV vector, can effectively achieve cardiac gene transfer and more stable expression after myocardial administration compared to AAV9, providing a new therapeutic strategy for CVD patients (57). Endothelial cells play a central role in the treatment of CVD. In a recent publication by Bozoglu et al., a significantly improved endothelial transduction rate was achieved with an rAAV coated with endothelial-affine peptides and polyamidoamine (PAMAMs) (58). Another novel strategy is to build a diverse *in vivo* targeting library by constructing AAV variants with Cap gene codon mutations, resulting in emergent synthetic variants with enhanced properties that can circumvent A20 mAb neutralizing effects (59). These improvements complement the deficiencies in AAV vectors and highlight the potential for the rational selection of AAV serotypes and capsid optimization in therapeutic strategies for CVD.

#### Take-off cardiovascular system—directional selection of restrictive promoters

In addition to identifying the most efficient myocardial-tissue-specific serotypes, another major factor to be considered is promoter selection. Promoters can be classified as constitutive (broad-spectrum type), tissue-restricted, and inducible, according to their expression patterns (60, 61). rAAV-based CVD gene therapy uses more constitutive promoters and specific gene expression regulatory elements to enable the expression of exogenous genes in specific tissue sites, which is more suitable for the therapeutic indication (10, 60, 62, 63). Many researchers have demonstrated these tissue-restricted promoters' utility and ability to enhance the specific expression of transgenes in the heart and vascular system; these include myocardium-specific promoters showing cardiac-selective expression and vascular-smooth-muscle-cell-specific and endothelial-cell-specific promoters.

The cardiovascular selective promoters currently developed include the cardiac troponin T promoter (cTnT) (64–67), atrial natriuretic factor (ANF) promoter (68), cardiac troponin T promoter (TNT4) (68), cardiac myocyte-specific cardiac troponin T (MYC) promoter (69), cardiac sodium-calcium exchanger (NCX1), and cardiac myosin light chain 2v (MLC-2v) promoter (70, 71), as well as the endothelial-specific Tie-2 promoter (72–74). Indeed, the rational selection of specific promoters is an alternative strategy to facilitate the development of *in vivo* gene delivery using rAAV-based therapies.

## Application of AAV vectors to gene therapy of cardiovascular disease

### Recombinant AAV and heart failure

#### Heart failure

HF, the leading cause of death from cardiovascular diseases, is a severe progressive disease that damages the heart and its function and irreversibly worsens without any therapeutic intervention (75, 76). When the heart's ejection capacity decreases, the body first activates a variety of compensatory mechanisms to maintain the heart's function at relatively normal levels (77), and these compensatory mechanisms play key roles in the development of HF. The pathophysiological changes are quite complex and can be summarized as three aspects: abnormal hemodynamics, the activation of neurohumoral regulation mechanisms, and ventricular remodeling (54, 77–80). Therefore, therapeutics based on the above three aspects are key to the treatment of HF.

#### rAAV vector was used for gene therapy in heart failure model

Understanding the pathological mechanisms and natural history of HF is the basis for achieving desired treatment outcomes. According to a large number of randomized controlled trials (Table 2), interventions that improve left ventricular systolic function are the main treatment modalities. In recent years, a better understanding of the pathogenesis of HF has led to a flurry of clinical trials. A prevalent clinical strategy for rAAV gene therapy in patients with severe HF is left ventricular function improvement and remodeling by driving the sarcoplasmic reticulum calcium ATPase (SERCA2a) pump to restoring calcium cycling and reduce ventricular rhythmic abnormalities in HF patients (103–105). SERCA2a strictly regulates myocardial contractility and mainly controls the transport of cytosolic calcium ions to the sarcoplasmic reticulum of cardiomyocytes (106, 107). In large animal models, AAV2/1-mediated hSERCA2a gene delivery *via* percutaneous circulation maximized SERCA2a expression and duration of exposure, significantly improved left ventricular function in a sheep model of HF, and showed no adverse outcomes for myocardial fibrosis or myocardial necrosis (108). While in CUPID phase 1/2a clinical trial, the safety of AAV1/SERCA2a for advanced HF was confirmed to be good, and the body function of most patients was improved; although, due to pre-existing NAbs in the body, two patients failed to improve (109). This also implies that it is urgent that we develop rAAV gene therapy that circumvents NAbs. With the exception of levosimendan (a Ca-sensitizer), treatment of HF patients focuses on reducing afterload, modification of cardiac remodeling, and other mechanisms.

**Table 2:** Preclinical application of AAV in cardiovascular disease.

**Product**	**Gene/regulator**	**Vector** **serotype**	**Indication/** **disease**	**Mechanism of action**	**Dose**	**Delivery method**	**Model**	**Reference**
AAV-hTAZ	TAZ	AAV9	Barth syndrome	Restores cardiolipin remodeling	2 × 10^10^ vg/g/ 1 × 10^10^ vg/g	Subcutaneous injection/ intravascular (retro-orbital) injection	TAZ-KO and TAZ-CKO models	(81)
AAV-hpNFAT dODN	dODN	AAV9	Cardiac hypertrophy and heart failure	Neutralizes NFAT and inhibits transcription of target genes	10^5^ vg/cell	Intravenous injection	Mouse model of cardiac hypertrophy	(82)
AAV-S100A1	S100A1	AAV9	HF	Restores sarcoplasmic reticulum calcium Ca^2+^ handling	1.5 × 10^13^vp	Retrograde coronary venous delivery	Postischemic porcine HF model	(83)
BNP116.I-1c	I-1c	AAV2-8	HF	Inhibits protein phosphatase activity and increases SERCA2a activity	1.0 × 10^13^ vg/3.0 × 10^12^ vg	Intracoronary injection	Swine model of ischemic HF	(84)
AAV9.SRF	SRF	AAV9	HF	Promotes cardiac myocyte growth	1011 vg /10^12^ vg	Intraperitoneal/intravenous injection	Mouse models of concentric and eccentric disease	(69)
AAV-miRNA-182	miRNA-182	AAV	HF	Downregulates PDCD4 and PACS2 to inhibit myocardial apoptosis	2 × 10^11^ vg	Left ventricular injection	Rat HF model	(85)
AAV9-shHRC	shHRC	AAV9	HF	Binds to TRN and SERCA to disrupt Ca2+ homeostasis in SR	10^10^vp	Tail vein injection	Mouse models of TAC-FH	(86)
AAV9.SERCA2a	SERCA2a	AAV9	HF	Improves systolic and diastolic function of the failing ventricle	1 × 10^12^ vg	Tail vein injection	Rat pressure-overload model	(87)
AAV9-VEGF-B 167 cDNA	VEGF-B 167	AAV2-9	HF	Attenuates oxidative stress and apoptosis	0.5 × 10^11^ vg	Intramuscular injection	DCM dog model	(88)
AAV9-PDE4B	PDE4B	AAV9	HF	Degrades cAMP, decreases cardiac contractile function, blunts β-AR responses	10^12^ vp/mouse	Unknown	Chronic iso model	(89)
AAV-FTO	FTO	AAV9	HF, MI	Regulates M6A, decreases cardiomyocyte contractile function	Unknown	Unknown	Mouse models of MI	(90)
AAV6-βARKct	βARKct	AAV6	HF	Inhibit GRK2 activation and improves βAR signaling	Unknown	Intramyocardial injection	Post-MI HF rats	(91)
rAAV6-caPI3K	PI3K	AAV6	HF	Modulates exercise-induced cardioprotection	2 × 10^11^vg	Tail vein injection	TAC model	(92)
AAV6:MCAD	MCAD	AAV6	TAC	Induces physiological cardiac growth and prevents pathological remodeling	2 × 10^11^vg	Tail vein injection	TAC model	(93)
AAV9-anti-miR-199a	Anti-miR-199a	AAV9	Cardiac hypertrophy and HF	Improves cardiac hypertrophy and restores cardiac function	1 × 10^11^vg	Subcutaneous injection	Cardiac hypertrophy model	(94)
AAV9-S15D-RLC	S15D-RLC	AAV9	HCM	Regulates striated muscle contraction	1.4 × 10^11^vg	Left ventricular cavity injection	Transgenic humanized WT-RLC and D166V mice	(55)
AAV9-cTnT-Klf5	KLF5	AAV9	DCM	KLF5 promotes myocardial ceramides accumulation	7 × 10^11^vp	Retroorbital injection	Animal models of diabetes	(65)
AAV-cTNT-CNTF-P2A-EGFP	CNTF	AAV9	DCM	Exacerbates cell apoptosis and cardiac fibrosis	5 × 10^11^ vg	Intraperitoneal injection	Type 1 Diabetic Models	(95)
AAV9-DNSUN1	DNSUN1	AAV2/9	DCM	Breaks LINC by disrupting SUN1 and protects cardiomyocytes from contraction-induced stress	5 × 10^10^vg	Thoracic cavity injection	LmnaF/Fmice	(96)
AAV9-miRNAi-LRP6	miRNAi-LRP6	AAV9	Loss or dysfunction of cardiac muscle cells	Lrp6 deficiency induces cell cycle activation in mouse hearts	Neonatal mice:8 × 10^9^/g; Adult mice:4 × 10^11^vg	Intraperitoneal injection	MI mices	(97)
AAV9-U7-AON5+6	U7-AON5+6	AAV9	HCM	Efficiently induces skipping of exons 5 and 6 of *Mybpc3* and increases *Var-4* mRNA level	2 × 10^11^vg	Tail vein injection	Mybpc3-targeted knock-in mouse model of HCM	(98)
AAV9-Cas9-gE51	DMD exon 51	AAV9	DMD	Induces expression of shortened dystrophin (DMDΔ 51-52) and improves skeletal muscle function	2 × 10^14^ vg/kg	Intravenous injection	DMD pig model	(99)
AAV9-sgRNA-51	DMD exon 51	AAV9	DMD	Restores dystrophin expression and assembly of DGC in dystrophic muscles	1.2 × 10^13^ vg	Intramuscular injection	Delta E50-MD dog model of DMD	(100)
AAV-CRISPR/Cas9-AID	AID	AAV9	DMD	Induces cytidine deaminase exon hopping to restore DMD protein expression	1.1 × 10^12^ vg	Intraperitoneal injection	DmdE4* mouse model	(101)
AAV-ΔR4-MD	MD	AAV9	DMD	Restore DGC and strengthens sarcolemma integrity in the MDX mouse heart	10^10^ vg	Cardiac cavity injection	MDX mouse model	(102)

TAZ, Tafazzin; TAZ-KO, Murine germline Taz-knockout models; TAZ-CKO, cardiac-specific Taz-knockout models; NFAT, nuclear factor of activated T cells; dODN, decoy oligodeoxynucleotides; I-1c, constitutively active inhibitor-1; SRF, serum response factor; PDCD4, programmed cell death4; PACS2, phosphoacidic cluster sorting protein; HRC, histidine-rich calcium binding protein; TRN, triadin; SR, sarcoplasmic reticulum; TAC-FH, transverse aortic constriction-induced failing heart; SERCA2a, sarcoplasmic reticulum calcium ATPase; VEGF-B, vascular endothelial growth factor-B; DCM, dilated cardiomyopathy; PDE4B, phosphodiesterase 4B; FTO, fat mass and obesity-associated; MI, myocardial ischemia; βAR, β-adrenergic receptor; GRK2, G protein–coupled receptor kinase 2; PI3K, phosphoinositide 3-kinase; TAC, transverse aortic-constriction; MCAD, medium chain acyl-coenzyme A dehydrogenase; miR, microRNAs; RLC, myosin regulatory; HCM, hypertrophic cardiomyopathy; KLF5, Krüppel-like factor 5; DCM, Diabetic cardiomyopathy; CNTF, ciliary neurotrophic factor; DNSUN1, dominantly negative acting sun1 miniprotein; LRP6, low-density lipoprotein receptor-related protein 6; AON, antisense oligoribonucleotides; U7-AON5+6, U7snRNA carrying AON sequences; Mybpc3, myosin-binding protein C; DMD, duchenne muscular dystrophy; AID, activation-induced cytidine deaminase; DGC, dystrophin-glycoprotein complex; MD, microdystrophin.

In rodent models, activation of the calcineurin-NFAT signaling pathway controls the hypertrophic growth of the myocardium, ultimately leading to heart failure. Remes et al. used AAV9 to express decoy oligodeoxynucleotides (dODN) to neutralize NFATc1-c4 in cardiomyocytes and reduce NFAT transcriptional activity, thereby preserving cardiac function during stress overload in mouse models of severe HF (82). Tafazzin (TAZ) is a mitochondrial protein associated with the inner mitochondrial membrane and is required for normal cardiolipin biosynthesis. Mutations in the TAZ gene cause Barth syndrome, but AAV9-hTZA gene therapy successfully reversed novel dysfunction in a cardio-specific Taz-knockout mouse model (81). A recent study demonstrated that AAV-JP2NT entry into the myocardium attenuated HF development and stress-induced transcriptional remodeling (110).

### Recombinant AAV and cardiomyopathy

#### Cardiomyopathy

Cardiomyopathy is a heterogeneous disease that results in systolic and diastolic dysfunction of the heart due to pathological changes to the myocardium (111). Most cardiomyopathies are nonhereditary, e.g., alcoholic cardiomyopathy, chemotherapy-related cardiomyopathy, infection-induced cardiomyopathy, and secondary cardiomyopathy linked to autoimmune disease. However, in most epidemiological studies, the genetic-linked diversity of cardiomyopathy types, such as hypertrophic cardiomyopathy (HCM), restrictive cardiomyopathy (RCM), dilated cardiomyopathy (DCM) and diabetic cardiomyopathy (DbCM), is still a complex issue (55, 102). Existing evidence implies that most myocardial diseases are caused by related pathogenic gene mutations. These genes are mainly involved in encoding cell structure and related functional proteins, and most are genes encoding sarcomeric proteins that have significant effects on cardiac function. In addition, several genes encoding cytoskeleton components and related connexins were also identified (111–113). Available data suggest that gene variants encoding sarcomere proteins may individually or collectively affect cardiac function, even if they do not cause myocardial disease.

#### Gene therapy for cardiomyopathy using rAAV vectors

Repairing and improving the systolic and diastolic functions of the heart is a long-term effective strategy against cardiomyopathy, and multiple gene therapies have been used to improve cardiac capacity, including cardiac regeneration, improving skeletal muscle function, and reducing cardiac hypertrophy (97, 99–101, 111). The current findings show that rAAV9 reversed the effects of HCM caused by the D166V mutation by delivering S15D-RLC into the hearts of humanized transgenic HCM-D166V mice, significantly improving intact cardiac function. Interestingly, rAAV9-S15D-RLC therapy enhanced systolic and diastolic function, but did not alter functioning WT-RLC (55). Mutations in the LaminA gene cause DCM, and delivery of Dominantly Negative acting Sun1 miniprotein (DNSUN1) using AAV9 significantly improved the progression of DCM and delayed HF, resulting in at least a five-fold extension in lifespan in mice that develop LaminA-induced DCM. This is because DNSUN1 protects cardiomyocytes from contraction-induced stress by disrupting SUN1 to disrupt the SUN-KASH LINK complex (96). The retro-orbital delivery of AAV9-cTnT-Klf5, an important transcription factor Krüppel-like factor 5 (KLF5), during cardiovascular remodeling directly binds the NADPH oxidase 4 (NOX4) promoter, and induction of NOX4 expression leads to oxidative stress and promotes the accumulation of myocardial ceramides, leading to DbCM (65). This suggests that the pharmacological or genetic inhibition of KLF5 may alleviate oxidative stress and improve myocardial function in diabetic mice. Combinations of gene therapy and antioxidants are promising new ideas for DbCM treatment. Martinez et al. investigated the effects of the systemic delivery of muscle A-kinase anchor protein β (mAKAPβ) in mouse models of myocardial infarction. The systemic administration of AAV9sc.shmAKAP (a novel self-complementary serotype 9 AAV expressing short-hairpin RNA of mAKAPβ under the control of a cardiomyocyte-specific promoter) inhibited mAKAPβ expression in cardiomyocytes and promoted the long-term stable recovery of the left ventricular ejection fraction, providing conceptual support for mAKAPβ being a therapeutic target for ischemic cardiomyopathy (114).

### Metabolic disease, atherosclerosis, and peripheral vascular diseases

Many patients with vascular disease still respond poorly to traditional drug treatments, and current medical therapies are not suitable for all CVD patients. Over the past 20 years, some promising CVD therapy developments have been seen, ranging from plasmid therapy to viral-mediated gene therapy. In particular, studies targeting major complications in CVD, including metabolic and genetic-related diseases such as hyperglycemia, hypercholesterolemia, and atherosclerosis, have yielded encouraging results (115). In recent sophisticated analyses conducted in randomized controlled trials, overexpression of the acquired functional variant proprotein convertase subtilisin/kexin type 9 (PCSK9) induced atherosclerosis in mice. In addition, targeting the low-density lipoprotein receptor (LDLR) with an integrated AAV-CRISPR vector and liver-directed destruction of LDLR was demonstrated to be a viable and reliable alternative way to study atherosclerosis (115, 116). AAV-directed LDLR gene therapy reduced plasma cholesterol levels in some patients in two clinical trials (from https://clinicaltrials.gov/; NCT02651675 and NCT04148001). Therefore, some patients with dyslipidemia or even atherosclerosis may benefit from AAV-mediated LDLR therapy and improvements in their apolipoprotein levels. Conversely, lowering serum low-density lipoprotein cholesterol in hyperlipidemia animal models was an effective strategy for reducing atherosclerotic CVD. The role of SIRT6 was also demonstrated by a preclinical study that involved creating a carotid plaque mouse model. In short, SIRT6 inhibits HIF-1α degradation to stimulate angiogenesis-related signaling pathways, revealing the cardiovascular risk associated with SIRT6 expression and suggesting that SIRT6 knockout is a feasible route to inhibit the development of atherosclerosis (117).

## Conclusion and future prospects

In brief, rAAV gene therapies provide the new opportunities for CVD patients, targeted therapy for refractory diseases is likely to have a far-reaching impact, which also implies that the optimization of viral capsid protein sequences and promoters will be a promising research direction in future preclinical trials. These strategies may better reap the potential benefits of rAAV gene therapy and more adequately address the distress and troubles of CVD patients.

## Author contributions

HZ designed, conceived, and wrote the manuscript. QZ reviewed the literature and supervised the manuscript. BH provided suggestions for manuscript revision. YW participated in the revision and polishing of the manuscript. XW participated in the revision and funding. All authors contributed to the article and approved the submitted version.

## Funding

This study was supported by Hangzhou Science and Technology Bureau (20201203B44), Public Welfare Technology Project of Zhejiang Province (LGF21H160033), and the Grant for 521 talent project of ZSTU.

## Conflict of interest

The authors declare that the research was conducted in the absence of any commercial or financial relationships that could be construed as a potential conflict of interest.

## Publisher's note

All claims expressed in this article are solely those of the authors and do not necessarily represent those of their affiliated organizations, or those of the publisher, the editors and the reviewers. Any product that may be evaluated in this article, or claim that may be made by its manufacturer, is not guaranteed or endorsed by the publisher.
